# Do invasive fungal infections affect kidney transplantation outcomes?

**DOI:** 10.3389/fcimb.2026.1982586

**Published:** 2026-10-27

**Authors:** Rong Ma, Dahao Zhang, Ziyang Luo, Junjie Sun, Ningzi Sun, Chunyan Liang, Chao Hu, Haibin Li, Liugen Lan, Jihua Wu, Jianhui Dong, Jiqiu Wen, Xuyong Sun, Ning Wen

**Affiliations:** 1 Institute of Transplant Medicine, The Second Affiliated Hospital of Guangxi Medical University, Nanning, China; 2 Guangxi Clinical Research Center for Organ Transplantation, Nanning, China; 3 Guangxi Key Laboratory of Organ Donation and Transplantation, Nanning, China

**Keywords:** acute rejection, allograft failure, invasive fungal infection, kidney transplantation, mortality

## Abstract

**Background:**

Invasive fungal infection (IFI) is a serious complication after kidney transplantation, but its longitudinal association with transplant outcomes remains unclear. We evaluated longitudinal associations of post-transplant IFI with subsequent outcomes, including how this association varied over time and by onset timing and IFI type.

**Methods:**

We conducted a single-center retrospective cohort study of 752 adult kidney transplant recipients transplanted in 2020–2023 and followed through December 31, 2025. First post-transplant IFI was adjudicated using standardized definitions and modeled as a time-varying exposure. The primary outcome was the composite outcome of all-cause mortality or graft failure; secondary outcomes were all-cause mortality, death-censored graft failure, and acute rejection. Analyses included period-specific incidence-rate comparisons, an adjusted Cox model allowing the IFI-associated HR to vary over post-transplant time, sequential risk-set matching, and competing-risk analyses.

**Results:**

IFI occurred in 137 recipients (18.2%) at a median of 284 days (IQR, 132–366) after transplantation; *Pneumocystis jirovecii* pneumonia (PJP) accounted for 99 cases (72.3%) and invasive aspergillosis (IA) for 29 (21.2%). Incidence rates of the composite outcome of all-cause mortality or graft failure were higher during IFI-exposed than non-IFI person-time within 6 months after transplantation (IRR, 34.05; 95% CI, 15.80–71.64) and after 6 months (IRR, 8.80; 95% CI, 4.89–16.00; both P < 0.001). The adjusted Cox model showed a decreasing IFI-associated HR over post-transplant time (P for time variation = 0.002). IFI was also associated with all secondary outcomes. In the multivariable matched-cohort model, IFI remained associated with the composite outcome (adjusted HR, 10.83; 95% CI, 5.51–21.29; P < 0.001). Within-IFI analyses showed higher risks of the composite outcome and all-cause mortality with IFI onset ≤6 versus >6 months after transplantation and with IA versus PJP.

**Conclusions:**

Post-transplant IFI was associated with adverse transplant outcomes, with a stronger association earlier in the post-transplant period. Among recipients with IFI, onset within 6 months after transplantation versus later onset and IA versus PJP were associated with poorer outcomes. These findings warrant multicenter validation.

## 1 Introduction

Maintenance immunosuppression after kidney transplantation reduces the risk of rejection but increases susceptibility to opportunistic infections (Pappas et al., 2010; van Delden et al., 2020). The reported incidence of IFI among kidney transplant recipients ranges from approximately 1% to 5% (Pappas et al., 2010; Patel et al., 2017; Hosseini‐Moghaddam et al., 2020; Li et al., 2025). Although the overall incidence is modest, reported proportions of graft failure and all-cause mortality after IFI range from 9.8% to 25% and from 31% to 61%, respectively (López-Medrano et al., 2016b; Patel et al., 2017; Hosseini‐Moghaddam et al., 2020). Few studies have accounted for the timing of IFI when evaluating its longitudinal association with subsequent transplant failure (Li et al., 2025; Eichenberger et al., 2026).

Large population-based and transplant-registry studies have reported associations of IFI and other post-transplant infections with death or graft failure (Hosseini‐Moghaddam et al., 2020; Jackson et al., 2021). However, mixed-organ populations, reliance on administrative coding, and broad infection definitions limit the applicability of these findings to clinically adjudicated IFI. Two United States Renal Data System (USRDS) studies reported associations of invasive aspergillosis (IA) and *Pneumocystis jirovecii* pneumonia (PJP), respectively, with higher risks of death and graft failure among kidney transplant recipients (Friedman et al., 2022; Mendoza et al., 2026), but each examined a single pathogen and relied on administrative diagnostic codes. More recent studies have used time-varying methods or propensity-score matching to preserve the temporal order between infection and outcomes, yet longitudinal evidence remains limited in kidney transplant cohorts with clinically adjudicated IFI and varying pathogen distributions (Li et al., 2025; Eichenberger et al., 2026).

The clinical consequences of IFI may differ by onset timing and IFI type. Predominant pathogens vary across cohorts and transplant periods, while individual pathogens may differ in timing, infection site, and clinical severity (Pappas et al., 2010; van Delden et al., 2020; Li et al., 2025). Existing longitudinal studies suggest that IFI timing may be associated with post-IFI outcomes, but comparisons across pathogens remain limited by sample size and pathogen composition (Li et al., 2025). During IFI treatment, immunosuppression often requires reassessment based on infection severity, antifungal drug interactions, and the recipient’s clinical status (Baddley et al., 2019; Sigera and Denning, 2023). Changes in immunosuppression may alter the balance among infection control, graft function, and rejection risk. Acute rejection is therefore an important post-IFI outcome to evaluate, although the interplay among IFI, adjustments to immunosuppression, and subsequent rejection has not been adequately characterized (Li et al., 2025).

To address these gaps, we conducted a single-center retrospective cohort study of adult kidney transplant recipients using standardized clinical definitions of post-transplant IFI. Our primary objective was to characterize the longitudinal association between post-transplant IFI and the composite outcome of all-cause mortality or graft failure, including how the strength of this association varied over post-transplant time and after IFI diagnosis. We also evaluated associations with all-cause mortality, death-censored graft failure, and acute rejection and explored whether post-IFI outcomes differed according to IFI onset timing and, among the two predominant IFI types, between PJP and IA.

## 2 Materials and methods

### 2.1 Study design and participants

This single-center retrospective cohort study used a census approach, screening all recipients who underwent kidney transplantation at our center between 2020 and 2023. Inclusion criteria were: (1) age ≥18 years at transplantation; and (2) at least 1 year of follow-up at our center, or less than 1 year of follow-up if a documented death or graft failure occurred within the first year after transplantation. Exclusion criteria were: (1) multiorgan transplantation involving the kidney; and (2) discontinuing follow-up at our center within 1 year after transplantation without a documented death or graft failure, such that subsequent IFI status and transplant outcomes could not be reliably ascertained.

All deceased-donor kidneys were allocated through the China Organ Transplant Response System. Living-related kidney donations were voluntary and non-remunerated and were approved by the Ethics Committee of the Second Affiliated Hospital of Guangxi Medical University. Organ donation and transplantation procedures complied with applicable laws, regulations, and ethical standards, and all required informed-consent procedures were completed. The study protocol was approved by the Ethics Committee of the Second Affiliated Hospital of Guangxi Medical University (approval number: 2026-KYC(138)).

### 2.2 IFI definition, exposure, and outcomes

The primary exposure was the first episode of IFI after kidney transplantation. Case definitions were based on the 2020 consensus definitions of the European Organisation for Research and Treatment of Cancer and the Mycoses Study Group Education and Research Consortium (EORTC/MSGERC) (Donnelly et al., 2019). IA, invasive candidiasis, and cryptococcosis were adjudicated according to the EORTC/MSGERC definitions, integrating relevant host factors, compatible clinical or radiographic findings, and pathogen-specific microbiological or histopathological evidence. PJP was adjudicated according to the EORTC/MSGERC framework, integrating relevant host factors, compatible clinical and radiographic findings, and supportive microbiological evidence (Lagrou et al., 2021). Because *P. jirovecii* PCR was not routinely performed at our center, supportive microbiological evidence included detection of *P. jirovecii* in a respiratory specimen by metagenomic next-generation sequencing (mNGS) or at least two consecutive serum β-D-glucan levels ≥80 pg/mL, after exclusion of alternative causes of β-D-glucan elevation. Neither mNGS detection nor β-D-glucan positivity was considered sufficient as a standalone diagnostic criterion; these microbiological findings were interpreted together with relevant host factors and clinical and radiographic findings.

All candidate cases were adjudicated by transplant physicians based on integrated clinical, radiographic, microbiological, and pathologic information. The diagnosis date was defined as the date on which an episode first met the prespecified criteria. The primary analyses included only proven or probable IFI. Possible IFI, colonization, contamination, superficial fungal infections, and isolated positive tests without a compatible clinical syndrome were excluded.

Fungal testing was performed when clinically indicated, including in the presence of compatible symptoms, radiographic abnormalities, or other findings raising clinical suspicion, rather than through routine screening of all kidney transplant recipients.

According to the center’s post-transplant prophylaxis protocol, TMP–SMX was planned for PJP prophylaxis and ganciclovir for CMV prophylaxis, each for 6 months. However, individual-level adherence to prophylaxis, dose adjustments, and discontinuation could not be reliably reconstructed from the electronic medical records and were not analyzed in this study.

Follow-up began at kidney transplantation and continued until death, graft failure, the last valid follow-up, or administrative censoring on December 31, 2025, whichever occurred first. Graft failure was defined as return to maintenance dialysis, repeat kidney transplantation, or documented irreversible loss of graft function.

For longitudinal analyses, IFI status was treated as a time-varying exposure: recipients contributed unexposed person-time before the diagnosis and exposed person-time thereafter.

The primary outcome was the composite outcome of all-cause mortality or graft failure. Secondary outcomes were all-cause mortality, death-censored graft failure, and acute rejection. Acute rejection included biopsy-proven rejection and clinically adjudicated rejection, the latter based on clinical presentation, ancillary testing, and treatment course.

### 2.3 Statistical analysis

Continuous variables were summarized as medians (interquartile ranges [IQR]) and compared using the Wilcoxon rank-sum test. Categorical variables were summarized as counts (percentages) and compared using Fisher’s exact test. The incidence rate of first IFI was calculated using observed person-time. The cumulative incidence of first IFI was estimated using the Aalen–Johansen estimator, treating all-cause mortality and graft failure occurring before the first IFI as competing events.

The full-cohort longitudinal analysis used transplantation as time zero. For the period-specific incidence-rate analysis, observed person-time was partitioned at 6 months after transplantation. Incidence rates during IFI-exposed and non-IFI person-time within and beyond 6 months were compared using exact Poisson methods, yielding incidence rate ratios (IRRs), 95% CIs, and P values. The primary analysis used a continuous-time Cox model to estimate the observational association between time-varying IFI status and the composite outcome of all-cause mortality or graft failure. To allow the IFI-associated hazard ratio (HR) to vary over post-transplant time, the model included an interaction between IFI status and a natural spline of log-transformed post-transplant time with 2 degrees of freedom. IFI status was incorporated as a time-varying exposure using start–stop intervals. Recipients who developed IFI contributed an unexposed interval from transplantation to the first IFI diagnosis and an exposed interval from the diagnosis date until the outcome or censoring; recipients who did not develop IFI contributed a single unexposed interval from transplantation until the outcome or censoring. Robust sandwich variance estimates were clustered by recipient to account for within-recipient correlation arising from multiple intervals. The model was adjusted *a priori* for recipient age, history of diabetes, donor age, and delayed graft function. The same time-varying Cox framework was used for the secondary outcomes, with outcome-specific definitions of events and censoring. In the all-cause mortality analysis, graft failure was treated as a censoring event; in the death-censored graft failure analysis, all-cause mortality was treated as a censoring event; and in the acute rejection analysis, both all-cause mortality and graft failure were treated as censoring events. These analyses therefore estimated cause-specific HRs.

Sequential risk-set matching was used for temporal validation of the primary full-cohort time-varying analysis. For each IFI case, the first IFI diagnosis date served as the index date. Each IFI case was matched to four control records from recipients whose transplantation dates were within ±30 days of the case’s transplantation date and who, at the case’s index date, were alive, had a functioning graft, had valid follow-up, and had not yet developed IFI. A recipient could contribute control records to multiple matched sets before their own IFI diagnosis. Control records retained their index-assigned control status and were not censored if the recipient subsequently developed IFI; recipients who later developed IFI could enter a matched set as cases using their own IFI diagnosis date as the index date.

For descriptive comparisons in the matched cohort, baseline variables were compared using conditional logistic regression stratified by matched-set identifier. Robust sandwich variance estimates were clustered by recipient identifier to account for repeated control records contributed by the same recipient. For outcome analyses, Cox models were stratified by matched-set identifier, with recipient-level robust sandwich variance estimates used to account for repeated records contributed by the same recipient. A summary HR over 0–365 days and interval-specific HRs for 0–90 and 91–365 days were estimated using a single piecewise time-varying Cox model including an interaction between IFI exposure and post-index time >90 days. For the composite outcome, cumulative probabilities over 365 days after the assigned index date were estimated as 1 minus the Kaplan–Meier survival estimate. The same analytical framework was applied to all-cause mortality, death-censored graft failure, and acute rejection using outcome-specific event and censoring definitions. Graft failure was treated as a censoring event in the all-cause mortality analysis, all-cause mortality as a censoring event in the death-censored graft failure analysis, and both all-cause mortality and graft failure as censoring events in the acute rejection analysis. These Cox models therefore estimated cause-specific HRs.

Complementary Fine–Gray subdistribution hazard models were fitted for the secondary outcomes using the same risk-set matched records, with the corresponding censoring events in the cause-specific Cox analyses treated as competing events. Models were stratified by matched-set identifier and used recipient-level robust sandwich variance estimates to account for repeated records contributed by the same recipient. Subdistribution hazard ratios (sHRs) were reported for 0–365, 0–90, and 91–365 days after the assigned index date. For the 91–365-day Fine–Gray analyses, a day-90 landmark risk set was used, restricted to records that remained free of the target and competing events and under observation at day 90. Fine–Gray models were not applied to the composite outcome because all-cause mortality and graft failure were both components of the endpoint rather than competing events. Cumulative incidence functions for the secondary outcomes over 365 days after the assigned index date were estimated using the Aalen–Johansen estimator with the same competing-event definitions. Covariates for the multivariable model of the composite outcome were selected *a priori* based on clinical relevance and prior literature.

Within the IFI cohort, exploratory unadjusted Cox models were used to compare early versus late IFI. Early IFI was defined as IFI occurring ≤6 months after transplantation, and late IFI as IFI occurring >6 months after transplantation. Because PJP and IA were the two predominant IFI types, an additional analysis compared 1-year post-IFI outcomes between recipients with PJP and those with IA. The same unadjusted Cox framework, with outcome-specific event and censoring definitions, was applied to the composite outcome of all-cause mortality or graft failure, all-cause mortality, death-censored graft failure, and acute rejection. Cumulative probabilities of the composite outcome were displayed using 1−Kaplan–Meier curves. HRs were reported as not estimable when no events occurred in one comparison group.

Longitudinal laboratory analyses used the first IFI diagnosis date as Day 0. Within non-overlapping windows around Days −90, −30, 0, 30, 90, and 180, the measurement closest to each target date was selected for eGFR, ALC, hemoglobin, serum albumin, and platelet count. At each time point, observed values were compared between early and late IFI and between PJP and IA using the Wilcoxon rank-sum test; nominal P values were reported without adjustment for multiple comparisons. All tests were two-sided, with P < 0.05 considered statistically significant; findings were interpreted in conjunction with effect estimates and 95% CIs. Descriptive analyses used variable-specific available denominators. Regression models were based on complete cases, and missing data were not imputed. All effect estimates were interpreted as observational associations rather than causal effects.

All statistical analyses were performed using R version 4.5.2 (R Foundation for Statistical Computing, Vienna, Austria).

## 3 Results

### 3.1 Study cohort and baseline characteristics

Between January 2020 and December 2023, 1,525 kidney transplantations were performed at our center. After excluding 77 multiorgan transplantations involving the kidney and 36 recipients aged <18 years, 1,412 adult kidney transplant recipients constituted the source cohort. Of these, 660 recipients were excluded from the analysis cohort (median follow-up, 68 days [IQR, 28–128 days]) because they discontinued follow-up at our center within 1 year after transplantation without prior death or graft failure, such that subsequent IFI status and outcomes could not be reliably ascertained. The remaining 752 recipients were included in the analysis. The selection process is shown in **Figure 1**, and cohort selection and follow-up completeness are summarized in **Supplementary Table S1**.

**Figure 1:**
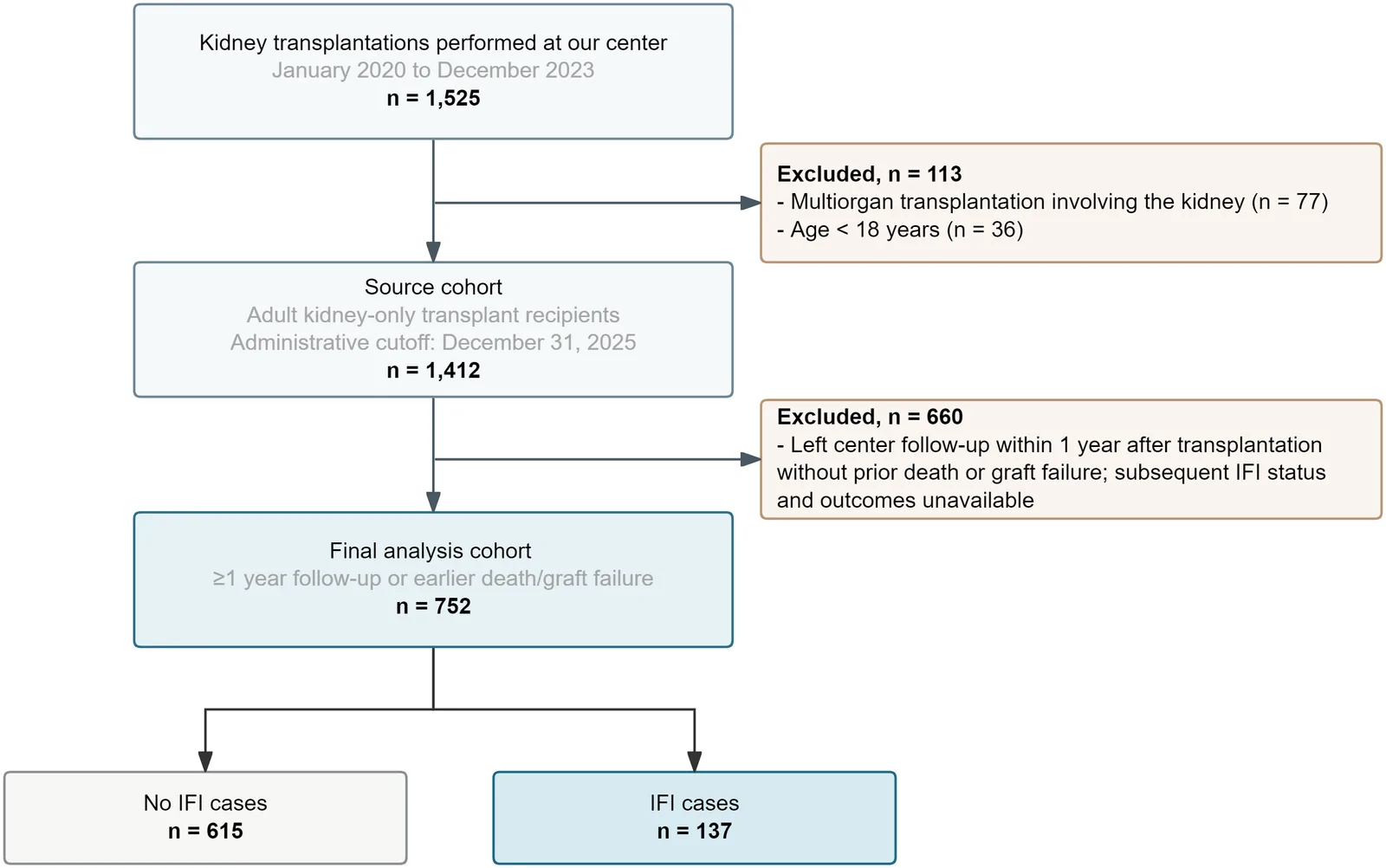
Cohort flow diagram.

Among the 752 recipients, the median follow-up was 937 days (IQR, 682–1,254 days). During follow-up, 137 recipients (18.2%) developed IFI, whereas 615 did not. Baseline characteristics were generally similar between groups. Compared with recipients without IFI, those who developed IFI had higher frequencies of delayed graft function (23.4% vs 14.1%, P = 0.010), perioperative reoperation (13.1% vs 4.9%, P = 0.001), and lymphatic leak (5.1% vs 1.0%, P = 0.004), longer perioperative hospital stays (13.0 [9.0–24.0] vs 10.0 [8.0–15.0] days, P < 0.001), and older donors (52.0 [45.0–59.0] vs 48.0 [37.0–56.0] years, P < 0.001) (**Table 1**).

**Table 1:** Baseline characteristics of kidney transplant recipients.

Clinical characteristics	Total (n = 752)	Non-IFI (n = 615)	IFI (n = 137)	P value
Recipient characteristics				
Recipient age, years	39.0 [32.0–48.0]	39.0 [32.0–48.0]	39.0 [33.0–49.0]	0.647
Recipient sex				0.355
Male	525 (69.8%)	434 (70.6%)	91 (66.4%)	
Female	227 (30.2%)	181 (29.4%)	46 (33.6%)	
Body mass index, kg/m²	21.8 [19.7–24.2]	21.8 [19.6–24.2]	21.8 [19.8–24.2]	0.915
Hypertension history	595 (79.1%)	491 (79.8%)	104 (75.9%)	0.298
Diabetes history	65 (8.6%)	51 (8.3%)	14 (10.2%)	0.501
Chronic liver disease history	134 (17.8%)	117 (19.0%)	17 (12.4%)	0.083
Coronary artery disease history	26 (3.5%)	20 (3.3%)	6 (4.4%)	0.449
Cerebrovascular disease history	23 (3.1%)	19 (3.1%)	4 (2.9%)	1.000
Smoking history	160 (21.3%)	126 (20.5%)	34 (24.8%)	0.299
Alcohol history	97 (12.9%)	75 (12.2%)	22 (16.1%)	0.259
Primary kidney disease				0.630
Glomerulonephritis	146 (19.4%)	123 (20.0%)	23 (16.8%)	
Diabetic kidney disease	26 (3.5%)	18 (2.9%)	8 (5.8%)	
Hypertensive nephropathy	36 (4.8%)	29 (4.7%)	7 (5.1%)	
Polycystic kidney disease	6 (0.8%)	5 (0.8%)	1 (0.7%)	
Alport syndrome	1 (0.1%)	1 (0.2%)	0 (0.0%)	
Other	3 (0.4%)	3 (0.5%)	0 (0.0%)	
Unknown	534 (71.0%)	436 (70.9%)	98 (71.5%)	
Dialysis modality				0.651
Hemodialysis	595 (79.1%)	491 (79.8%)	104 (75.9%)	
Hemodialysis + peritoneal dialysis	24 (3.2%)	20 (3.3%)	4 (2.9%)	
Peritoneal dialysis	114 (15.2%)	89 (14.5%)	25 (18.2%)	
Preemptive transplantation	19 (2.5%)	15 (2.4%)	4 (2.9%)	
Dialysis duration, months	25.0 [14.0–43.0]	25.0 [14.0–43.0]	24.0 [13.0–41.0]	0.797
Transplant and perioperative characteristics				
Repeat kidney transplantation	8 (1.1%)	7 (1.1%)	1 (0.7%)	1.000
Operation duration, min	165.0 [144.0–187.0]	164.0 [144.0–187.0]	166.0 [143.0–190.0]	0.874
Delayed graft function	119 (15.8%)	87 (14.1%)	32 (23.4%)	0.010
Perioperative reoperation	48 (6.4%)	30 (4.9%)	18 (13.1%)	0.001
Urine leak	35 (4.7%)	25 (4.1%)	10 (7.3%)	0.116
Lymphatic leak	13 (1.7%)	6 (1.0%)	7 (5.1%)	0.004
Wound-healing disorder	16 (2.1%)	12 (2.0%)	4 (2.9%)	0.510
Graft ureteral stenosis	37 (4.9%)	28 (4.6%)	9 (6.6%)	0.380
Perioperative hyperamylasemia	117 (15.6%)	88 (14.3%)	29 (21.2%)	0.051
Perioperative hospital stay, days	11.0 [8.0–16.0]	10.0 [8.0–15.0]	13.0 [9.0–24.0]	<0.001
Immunosuppression				
Induction therapy				0.570
Basiliximab	394 (52.4%)	318 (51.7%)	76 (55.5%)	
ALG	195 (25.9%)	163 (26.5%)	32 (23.4%)	
rATG	28 (3.7%)	21 (3.4%)	7 (5.1%)	
Methylprednisolone alone	135 (18.0%)	113 (18.4%)	22 (16.1%)	
Initial maintenance regimen				1.000
Tacrolimus + MPA + prednisone	747 (99.3%)	611 (99.3%)	136 (99.3%)	
Other regimen	5 (0.7%)	4 (0.7%)	1 (0.7%)	
Immunologic and donor characteristics				
HLA mismatch points	3.0 [3.0–4.0]	3.0 [3.0–4.0]	3.0 [3.0–4.0]	0.183
Donor age, years	49.0 [38.0–57.0]	48.0 [37.0–56.0]	52.0 [45.0–59.0]	<0.001
Donor sex				0.244
Male	596 (79.3%)	482 (78.4%)	114 (83.2%)	
Female	156 (20.7%)	133 (21.6%)	23 (16.8%)	
Donor type				0.502
DBD	737 (98.0%)	604 (98.2%)	133 (97.1%)	
DCD	7 (0.9%)	5 (0.8%)	2 (1.5%)	
Living donor	8 (1.1%)	6 (1.0%)	2 (1.5%)	
Expanded-criteria donor	240 (33.1%)	189 (31.9%)	51 (38.6%)	0.152
Donor serum creatinine, μmol/L	100.0 [69.2–145.0]	97.0 [67.2–144.4]	107.5 [77.0–145.0]	0.206
Donor body mass index, kg/m²	23.4 [21.0–25.1]	23.4 [21.2–25.4]	22.9 [20.8–24.8]	0.258

ALG, anti-lymphocyte globulin; DBD, donation after brain death; DCD, donation after circulatory death; HLA, human leukocyte antigen; IFI, invasive fungal infection; MPA, mycophenolic acid; rATG, rabbit antithymocyte globulin.

### 3.2 Incidence and characteristics of post-transplant IFI

Among the 752 recipients, 1,822.9 person-years of observed follow-up yielded a first-IFI incidence rate of 75.2 per 1,000 person-years (95% CI, 63.1–88.8). With death and graft failure before the first IFI treated as competing events, the cumulative incidence of first IFI at 1, 2, 3, and 5 years after transplantation was 13.6% (95% CI, 11.1%–16.0%), 17.3% (14.6%–20.0%), 18.9% (16.0%–21.8%), and 19.3% (16.3%–22.2%), respectively (**Figure 2**). The median time to first IFI was 284 days after transplantation (IQR, 132–366 days); 51 episodes (37.2%) occurred ≤6 months after transplantation (early IFI), whereas 86 (62.8%) occurred >6 months (late IFI) (**Table 2**; **Supplementary Figure S1**). PJP was the most common IFI type (99 cases, 72.3%; diagnosed using a center-adapted framework based on EORTC/MSGERC criteria, with detailed clinical characteristics summarized in **Supplementary Table S2**), followed by IA (29, 21.2%), invasive candidiasis (6, 4.4%), cryptococcosis (2, 1.5%), and other IFI (1, 0.7%). PJP accounted for 81.4% of late versus 56.9% of early IFI, whereas IA accounted for 33.3% of early versus 14.0% of late IFI. Pulmonary involvement was present in 131 cases (95.6%); the full pathogen and infection-site distributions are shown in **Table 2**.

**Figure 2:**
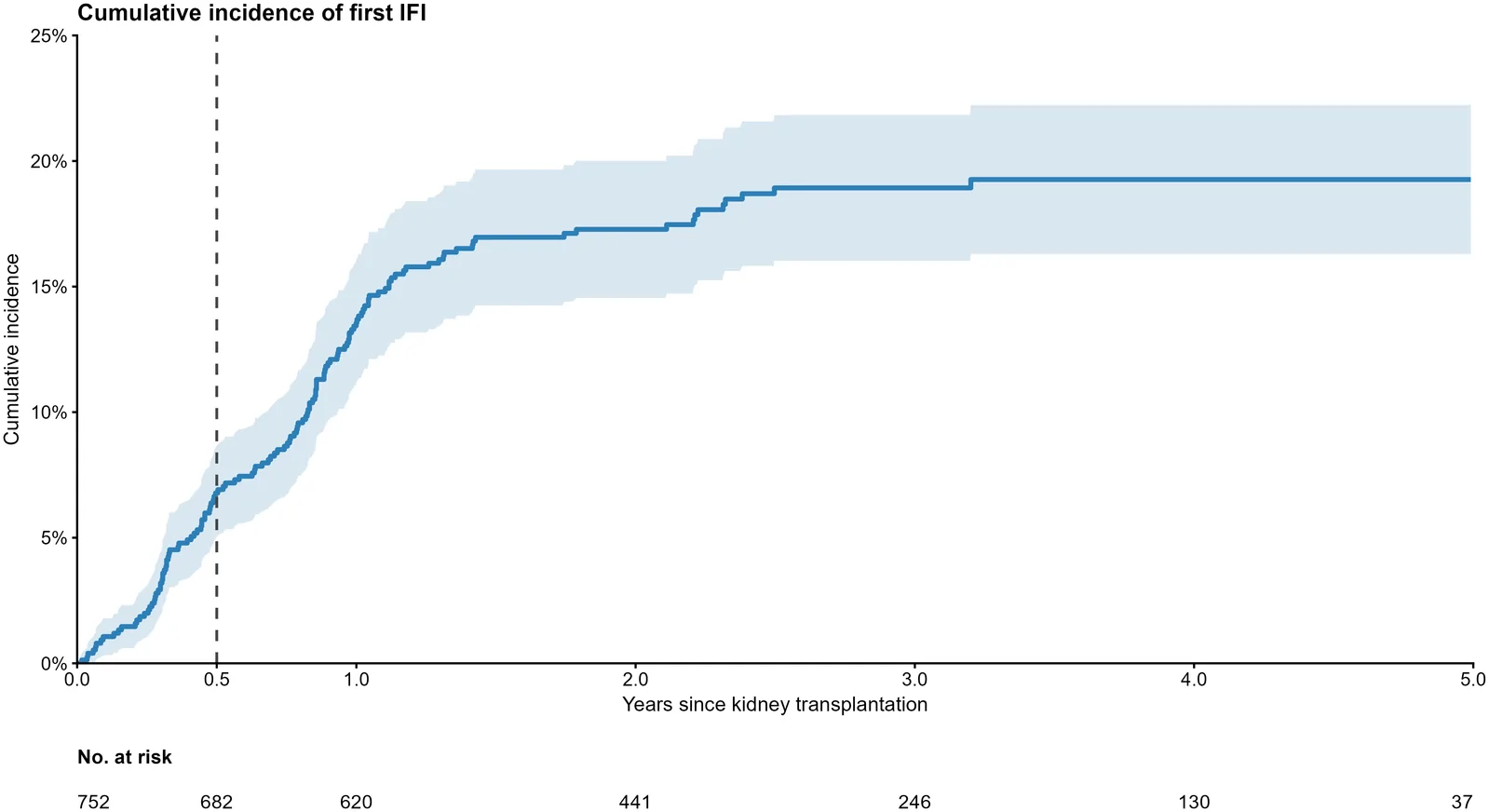
Cumulative incidence of first IFI. Death and graft failure before IFI were treated as competing events. The dashed line indicates the 6-month threshold used for early versus late IFI classification.

**Table 2:** Characteristics of first IFI episodes after kidney transplantation.

Characteristic	Total (n = 137)	Early IFI (≤6 months) (n = 51)	Late IFI (>6 months) (n = 86)
Time to first IFI, median [IQR], days	284 [132–366]	112 [80–151]	346 [296–415]
IFI type			
PJP	99 (72.3%)	29 (56.9%)	70 (81.4%)
IA	29 (21.2%)	17 (33.3%)	12 (14.0%)
IC	6 (4.4%)	2 (3.9%)	4 (4.7%)
Cryptococcosis	2 (1.5%)	2 (3.9%)	0 (0.0%)
Other	1 (0.7%)	1 (2.0%)	0 (0.0%)
Specific pathogen			
*Pneumocystis jirovecii*	99 (72.3%)	29 (56.9%)	70 (81.4%)
*Aspergillus* species			
*Aspergillus fumigatus*	27 (19.7%)	16 (31.4%)	11 (12.8%)
*Aspergillus flavus*	3 (2.2%)	1 (2.0%)	2 (2.3%)
*Candida* species			
*Candida albicans*	2 (1.5%)	0 (0.0%)	2 (2.3%)
*Candida glabrata*	2 (1.5%)	0 (0.0%)	2 (2.3%)
*Candida parapsilosis*	3 (2.2%)	2 (3.9%)	1 (1.2%)
*Candida tropicalis*	1 (0.7%)	1 (2.0%)	0 (0.0%)
*Cryptococcus* species	2 (1.5%)	2 (3.9%)	0 (0.0%)
Mucorales species	1 (0.7%)	1 (2.0%)	0 (0.0%)
Site of infection			
Pulmonary	131 (95.6%)	47 (92.2%)	84 (97.7%)
Bloodstream	7 (5.1%)	4 (7.8%)	3 (3.5%)
Intra-abdominal	2 (1.5%)	1 (2.0%)	1 (1.2%)
Genitourinary	2 (1.5%)	2 (3.9%)	0 (0.0%)
Central nervous system	1 (0.7%)	1 (2.0%)	0 (0.0%)
Other	1 (0.7%)	0 (0.0%)	1 (1.2%)

IFI types were mutually exclusive. Mixed infections (n = 4) and multisite involvement (n = 5) were present; pathogen and site-of-infection categories were not mutually exclusive, and percentages may therefore exceed 100%.

IA, invasive aspergillosis; IC, invasive candidiasis; IFI, invasive fungal infection; IQR, interquartile range; PJP, *Pneumocystis jirovecii* pneumonia.

### 3.3 Longitudinal associations of IFI with transplant outcomes

During follow-up, 84 events of the composite outcome of all-cause mortality or graft failure occurred. In the full-cohort continuous-time Cox spline model, adjusted for covariates selected *a priori* based on clinical relevance and prior literature, the association between IFI and the composite outcome varied significantly over post-transplant time, with an overall decreasing pattern (P for time variation = 0.002; **Figure 3**). In the 6-month piecewise incidence-rate analysis, the composite-outcome incidence rates during IFI-exposed and non-IFI person-time were 1.792 and 0.053 events per person-year, respectively, during the first 6 months (incidence rate ratio [IRR], 34.05; 95% CI, 15.80–71.64; P < 0.001), and 0.138 and 0.016 events per person-year, respectively, thereafter (IRR, 8.80; 95% CI, 4.89–16.00; P < 0.001; **Table 3**).

**Figure 3:**
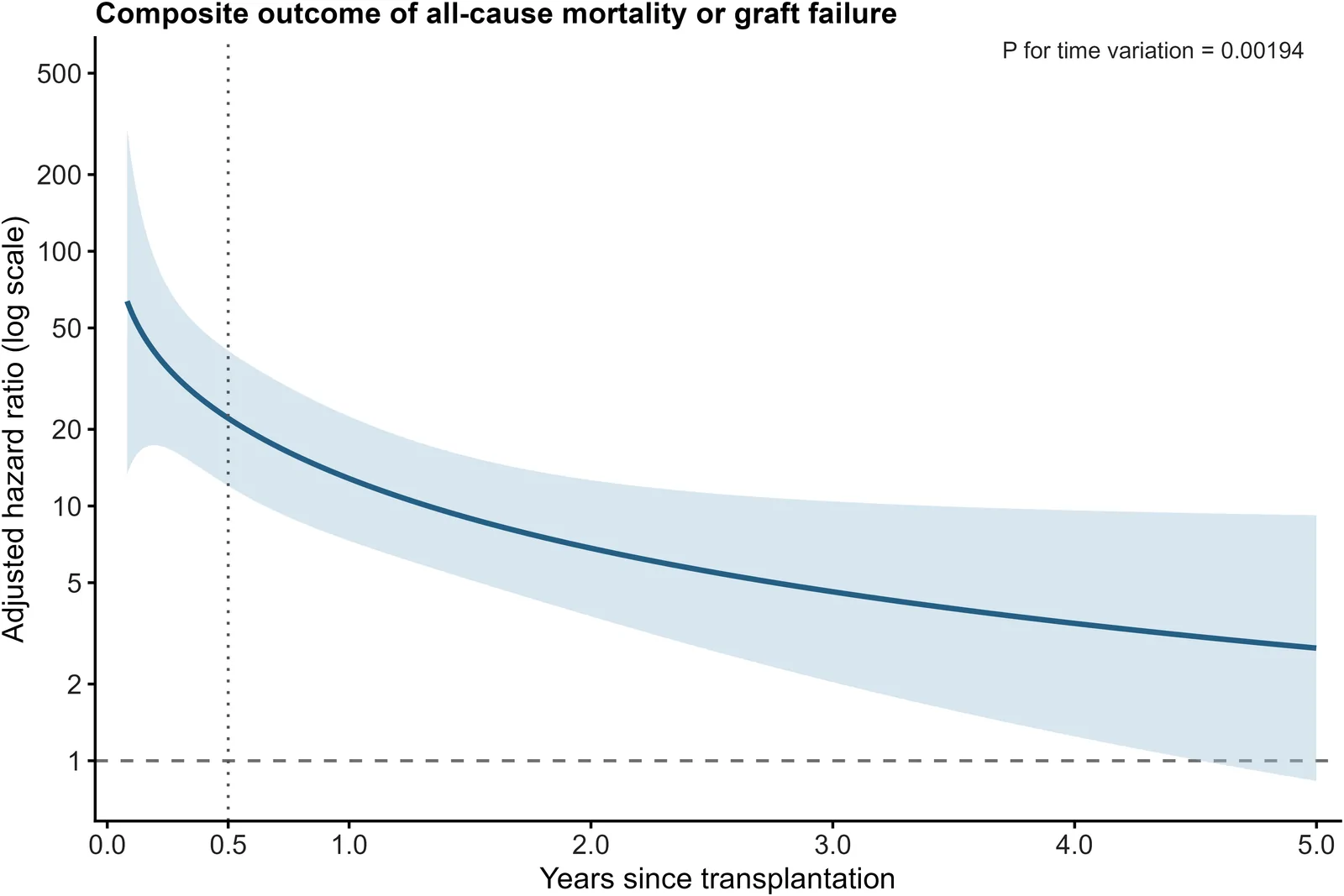
Adjusted time-varying hazard ratio for time-updated IFI status and the composite outcome of all-cause mortality or graft failure. The model was adjusted *a priori* for recipient age, history of diabetes, donor age, and delayed graft function.

**Table 3:** Outcome event counts and incidence rates during IFI-exposed and non-IFI person-time across post-transplant time intervals.

	Time since transplantation (months)	IFI outcome events, n	IFI IR (events/PY)	Non-IFI outcome events, n	Non-IFI IR (events/PY)	IRR (95% CI)	P value
The composite outcome of all-cause mortality or graft failure	≤6	14	1.792	19	0.053	34.05 (15.80–71.64)	<0.001
	>6	28	0.138	23	0.016	8.80 (4.89–16.00)	<0.001
All-cause mortality	≤6	13	1.664	13	0.036	46.22 (19.74–108.21)	<0.001
	>6	19	0.094	7	0.005	19.62 (7.90–55.24)	<0.001
Death-censored graft failure	≤6	1	0.128	6	0.017	7.70 (0.17–63.49)	0.139
	>6	9	0.045	16	0.011	4.07 (1.58–9.77)	0.002
Acute rejection	≤6	1	0.128	4	0.011	11.55 (0.23–116.76)	0.102
	>6	35	0.173	65	0.044	3.89 (2.50–5.96)	<0.001

CI, confidence interval; IFI, invasive fungal infection; IR, incidence rate; IRR, incidence rate ratio; PY, person-years.

The two component outcomes comprised 52 all-cause mortality events and 32 death-censored graft failure events. The adjusted cause-specific HR for all-cause mortality remained elevated throughout follow-up, without statistically significant time variation (P for time variation = 0.473; **Supplementary Figure S2A**); the corresponding IRRs during the first 6 months and thereafter were 46.22 (95% CI, 19.74–108.21; P < 0.001) and 19.62 (95% CI, 7.90–55.24; P < 0.001), respectively. For death-censored graft failure, the adjusted cause-specific HR showed an early increase followed by a decline, without statistically significant time variation (P for time variation = 0.139; **Supplementary Figure S2B**); the corresponding IRRs were 7.70 (95% CI, 0.17–63.49; P = 0.139) and 4.07 (95% CI, 1.58–9.77; P = 0.002), respectively. There were 105 acute rejection events during follow-up. The adjusted cause-specific HR for acute rejection generally declined over time, without statistically significant time variation (P for time variation = 0.097; **Supplementary Figure S2C**); the corresponding IRRs were 11.55 (95% CI, 0.23–116.76; P = 0.102) during the first 6 months and 3.89 (95% CI, 2.50–5.96; P < 0.001) thereafter (**Table 3**). Early estimates for death-censored graft failure and acute rejection were imprecise because only one event occurred during IFI-exposed person-time for each outcome during the first 6 months.

The risk-set matched analysis comprised 137 matched sets, each containing one IFI case and four eligible at-risk control records assigned the same index date as the corresponding case (137 IFI records and 548 matched control records; **Supplementary Table S3**). Within 1 year after the assigned index date, the composite outcome of all-cause mortality or graft failure occurred in 37 IFI records and 17 matched control records, with cumulative probabilities of 27.5% and 3.3%, respectively (**Figure 4**). IFI exposure was associated with a higher risk of the composite outcome over 0–365 days (HR, 11.20; 95% CI, 6.26–20.04; P < 0.001). The association was strongest during 0–90 days (HR, 21.19; 95% CI, 8.42–53.32; P < 0.001) and remained statistically significant during 91–365 days (HR, 2.72; 95% CI, 1.10–6.70; P = 0.030; **Table 4**).

**Figure 4:**
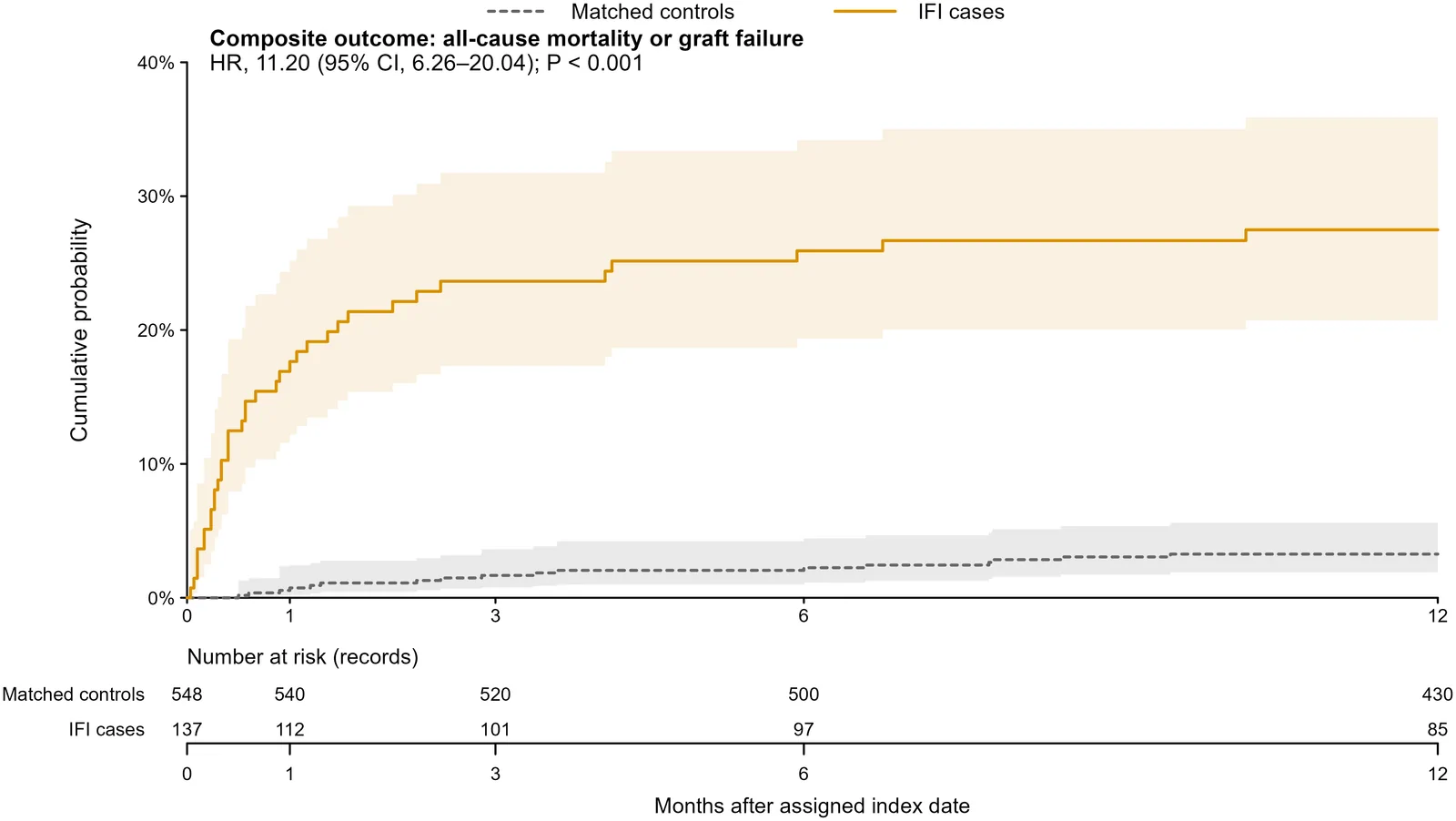
Cumulative probability of the composite outcome of all-cause mortality or graft failure after assigned index dates in IFI cases and risk-set matched controls.

**Table 4:** Time-specific associations of IFI with outcomes after assigned index dates in the risk-set matched cohort.

Outcome	Time after index date	Cox HR (95% CI)	P value	Fine–Gray sHR (95% CI)	P value
The composite outcome of all-cause mortality or graft failure					
	Overall 0–365 days	11.20 (6.26–20.04)	<0.001	—	—
	0–90 days	21.19 (8.42–53.32)	<0.001	—	—
	91–365 days	2.72 (1.10–6.70)	0.030	—	—
All-cause mortality					
	Overall 0–365 days	13.15 (6.49–26.67)	<0.001	13.24 (6.54–26.83)	<0.001
	0–90 days	21.42 (7.77–59.05)	<0.001	21.60 (7.84–59.50)	<0.001
	91–365 days	2.88 (0.94–8.84)	0.065	2.88 (0.94–8.84)	0.065
Death-censored graft failure					
	Overall 0–365 days	6.83 (2.55–18.27)	<0.001	5.48 (1.95–15.41)	0.001
	0–90 days	20.00 (2.84–140.82)	0.003	10.04 (2.45–41.19)	0.001
	91–365 days	2.67 (0.62–11.48)	0.188	2.51 (0.57–11.19)	0.226
Acute rejection					
	Overall 0–365 days	3.89 (1.73–8.74)	<0.001	3.40 (1.63–7.11)	0.001
	0–90 days	4.00 (1.82–8.79)	<0.001	3.50 (1.60–7.65)	0.002
	91–365 days	3.74 (0.97–14.36)	0.055	3.74 (0.97–14.36)	0.055

The composite outcome was not analyzed using a Fine–Gray model.

In cause-specific Cox analyses of the secondary outcomes, IFI exposure was associated with all-cause mortality over 0–365 days (HR, 13.15; 95% CI, 6.49–26.67; P < 0.001). The corresponding HRs were 21.42 (95% CI, 7.77–59.05; P < 0.001) during 0–90 days and 2.88 (95% CI, 0.94–8.84; P = 0.065) during 91–365 days, indicating a marked early association but less precise evidence thereafter (**Table 4**; **Supplementary Figure S3A**). For death-censored graft failure, the overall cause-specific HR was 6.83 (95% CI, 2.55–18.27; P < 0.001), with HRs of 20.00 (95% CI, 2.84–140.82; P = 0.003) during 0–90 days and 2.67 (95% CI, 0.62–11.48; P = 0.188) during 91–365 days (**Table 4**; **Supplementary Figure S3B**). For acute rejection, the overall cause-specific HR was 3.89 (95% CI, 1.73–8.74; P < 0.001), with HRs of 4.00 (95% CI, 1.82–8.79; P < 0.001) and 3.74 (95% CI, 0.97–14.36; P = 0.055) during 0–90 and 91–365 days, respectively (**Table 4**; **Supplementary Figure S3C**). Thus, for death-censored graft failure and acute rejection, the largest point estimates were observed during the first 90 days, although the early estimates were imprecise. Fine–Gray analyses showed similar overall associations, with sHRs of 13.24 (95% CI, 6.54–26.83; P < 0.001) for all-cause mortality, 5.48 (95% CI, 1.95–15.41; P = 0.001) for graft failure, and 3.40 (95% CI, 1.63–7.11; P = 0.001) for acute rejection. Interval-specific Fine–Gray estimates were broadly consistent with the corresponding cause-specific Cox results (**Table 4**).

Finally, in the multivariable model for the composite outcome of all-cause mortality or graft failure, covariates were selected *a priori* based on prior literature and clinical relevance and entered simultaneously into the model. IFI exposure remained associated with the composite outcome after multivariable adjustment (adjusted HR, 10.83; 95% CI, 5.51–21.29; P < 0.001). Diabetes history (adjusted HR, 3.79; 95% CI, 1.11–12.93; P = 0.034) and respiratory bacterial infection within 180 days before the assigned index date (adjusted HR, 5.58; 95% CI, 1.17–26.60; P = 0.031) were also associated with the composite outcome (**Table 5**; **Supplementary Table S4**).

**Table 5:** Multivariable analysis of the composite outcome of all-cause mortality or graft failure within 1 year after the assigned index date in the sequential risk-set matched cohort.

Variable	Adjusted HR (95% CI)	P value
IFI	10.83 (5.51–21.29)	<0.001
Recipient age, per 10 years	0.91 (0.64–1.30)	0.616
Diabetes history	3.79 (1.11–12.93)	0.034
Donor age, per 10 years	1.25 (0.95–1.65)	0.107
Delayed graft function	1.13 (0.44–2.89)	0.796
Acute rejection within 180 days before index	2.48 (0.49–12.66)	0.274
Respiratory bacterial infection within 180 days before index	5.58 (1.17–26.60)	0.031

### 3.4 Post-IFI outcomes by onset timing and IFI type

Within the IFI cohort, exploratory unadjusted Cox comparisons were performed over 365 days after the first IFI diagnosis. The composite outcome of all-cause mortality or graft failure occurred in 21 of 51 recipients with early IFI and 16 of 86 with late IFI. The 1-year cumulative probabilities of the composite outcome after IFI diagnosis were 41.3% (95% CI, 29.2%–56.0%) and 19.0% (95% CI, 12.1%–29.1%), respectively; the HR for early versus late IFI was 2.38 (95% CI, 1.24–4.57; P = 0.009; **Table 6**, **Supplementary Figure S4A**). All-cause mortality occurred in 17 of 51 recipients with early IFI and 13 of 86 with late IFI; the HR for early versus late IFI was 2.34 (95% CI, 1.14–4.82; P = 0.021; **Table 6**). The corresponding HRs for death-censored graft failure and acute rejection were 2.57 (95% CI, 0.57–11.48; P = 0.217) and 2.70 (95% CI, 0.85–8.51; P = 0.091), respectively; neither association reached statistical significance (**Table 6**). Recipients with early IFI had lower observed eGFR, ALC, and hemoglobin values at selected time points around IFI diagnosis (**Supplementary Figures S5**, **S6**).

**Table 6:** Post-IFI outcomes according to onset timing and IFI type.

Comparison	Outcome	HR (95% CI)	P value
Early vs late IFI	The composite outcome of all-cause mortality or graft failure	2.38 (1.24–4.57)	0.009
	All-cause mortality	2.34 (1.14–4.82)	0.021
	Death-censored graft failure	2.57 (0.57–11.48)	0.217
	Acute rejection	2.70 (0.85–8.51)	0.091
PJP vs IA	The composite outcome of all-cause mortality or graft failure	0.25 (0.12–0.49)	<0.001
	All-cause mortality	0.24 (0.11–0.52)	<0.001
	Death-censored graft failure	0.27 (0.06–1.20)	0.085
	Acute rejection	Not estimable	—

Early and late IFI were defined as occurring ≤6 and >6 months after transplantation, respectively.

CI, confidence interval; HR, hazard ratio; IA, invasive aspergillosis; IFI, invasive fungal infection; PJP, *Pneumocystis jirovecii* pneumonia.

Among 128 recipients with PJP or IA, the composite outcome of all-cause mortality or graft failure occurred in 17 of 99 PJP recipients and 16 of 29 IA recipients. The 1-year cumulative probabilities of the composite outcome after IFI diagnosis were 17.4% (95% CI, 11.2%–26.5%) and 55.9% (95% CI, 38.9%–74.3%), respectively; the HR for PJP versus IA was 0.25 (95% CI, 0.12–0.49; P < 0.001; **Table 6**, **Supplementary Figure S4B**). All-cause mortality occurred in 13 of 99 PJP recipients and 13 of 29 IA recipients; the HR for PJP versus IA was 0.24 (95% CI, 0.11–0.52; P < 0.001; **Table 6**). The HR for death-censored graft failure was 0.27 (95% CI, 0.06–1.20; P = 0.085), whereas the HR for acute rejection was not estimable because no acute rejection event occurred in the IA group (**Table 6**). Recipients with IA had lower observed eGFR, ALC, hemoglobin, and platelet counts than PJP recipients at selected time points before and after IFI diagnosis (**Supplementary Figures S7**, **S8**).

## 4 Discussion

In this cohort of 752 kidney transplant recipients, we evaluated the longitudinal associations of IFI with subsequent transplant outcomes. IFI occurred in 137 recipients (18.2%), with PJP predominating. In the primary longitudinal analysis, incidence rates of the composite outcome of all-cause mortality or graft failure were higher during IFI-exposed than non-IFI person-time both within and beyond 6 months after transplantation. The adjusted Cox model showed that the IFI-associated HR varied over post-transplant time with an overall decreasing pattern. Risk-set matching showed a concordant association that was strongest within the first 90 days after the assigned index date, and the overall association remained after multivariable adjustment. IFI was also associated with all-cause mortality, death-censored graft failure, and acute rejection. Exploratory within-IFI analyses showed higher risks of the composite outcome and all-cause mortality with early versus late IFI and with IA versus PJP.

Li et al. used a matched longitudinal approach and reported associations of IFI with the composite outcome of all-cause mortality or graft failure and all-cause mortality (Li et al., 2025); other cohorts have reported adverse outcomes after IFI or specific fungal infections (López-Medrano et al., 2016a; Seok et al., 2020a, Seok et al., 2020b; Mendoza et al., 2026). We modeled IFI as a time-varying exposure and used risk-set matching for temporal validation to preserve temporal ordering and avoid classifying pre-IFI person-time as exposed. Where applicable, competing risks were addressed using complementary cause-specific Cox and Fine–Gray models. Nevertheless, residual confounding cannot be excluded, and the observed associations should not be interpreted as causal.

The temporal findings reflected two related but distinct time scales. In the overall cohort, the association between IFI and the composite outcome was strongest earlier after transplantation and decreased over post-transplant time, whereas in the risk-set matched analysis, the association was strongest during the first 90 days after the assigned index date. Consistent with the former pattern, exploratory analyses restricted to recipients with IFI showed poorer post-IFI outcomes among those with onset within 6 months after transplantation than among those with later-onset IFI. Previous studies suggest that the timing of PJP may be associated with post-infection outcomes, but comparisons of early and late disease have not been consistent: some studies reported poorer outcomes with early PJP, whereas late PJP has also been associated with graft failure and death (Kaminski et al., 2020; Li et al., 2025; Konwai et al., 2026; Wang et al., 2026b). The stronger association earlier after transplantation may reflect greater recipient vulnerability during a period of greater immunosuppressive burden, postoperative complications, graft instability, and concurrent infections (van Delden et al., 2020; Bharati et al., 2023). The stronger association during the first 90 days after the assigned index date may additionally reflect the acute clinical consequences of IFI and its treatment, including organ dysfunction and adjustments to immunosuppression. These temporal patterns should not be interpreted as causal effects of timing, because infection severity, graft status, prior treatment, and immune status may differ across periods.

The IFI burden and pathogen distribution in our cohort differed from those reported in other kidney transplant cohorts. A prospective five-center German cohort of 1,258 recipients reported a 6.7% cumulative incidence of fungal infection, with *Candida* and *Aspergillus* predominating, PJP accounting for 13.5%, and mortality highest among patients with IA (Schröter et al., 2026). TRANSNET and the study by Li et al. also reported predominantly *Candida*- or mold-associated IFI (Pappas et al., 2010; Li et al., 2025). In contrast, IFI occurred in 18.2% of recipients in our cohort, and PJP accounted for 72.3% of IFI cases. This difference may partly reflect differences in diagnostic practices. Fungal testing at our center was clinically indicated rather than routine, and PJP adjudication used respiratory mNGS as supportive, rather than standalone, microbiological evidence because *P. jirovecii* PCR was not routinely performed at our center. Recent kidney transplant studies have also reported mNGS-based diagnostic applications for PJP and other fungal infections (Zhang et al., 2025; Wang et al., 2026a). In addition, exclusion of recipients with early loss to follow-up and unascertainable subsequent IFI status may have influenced the observed IFI incidence. Differences in incidence and pathogen composition across centers should therefore be interpreted in the context of case definitions, diagnostic strategies, and follow-up practices.

Among recipients with IFI, those with early-onset IFI also had lower observed eGFR, ALC, and hemoglobin values at selected time points around IFI diagnosis. These differences may reflect poorer graft function and greater systemic or immune compromise surrounding early-onset IFI, but the longitudinal laboratory findings were exploratory and should be interpreted as descriptive clinical phenotypes rather than evidence of specific causal mechanisms.

Within the IFI cohort, recipients with IA had higher risks of the composite outcome and all-cause mortality than those with PJP and had lower observed eGFR, ALC, hemoglobin, and platelet counts at selected time points. Previous kidney transplant studies have generally examined PJP and IA separately or reported pathogen-specific outcomes without a formal head-to-head comparison. Both infections have been associated with mortality and graft failure, and a recent prospective multicenter cohort reported the highest mortality among recipients with IA (López-Medrano et al., 2016a; Seok et al., 2020b; Friedman et al., 2022; Mendoza et al., 2026; Schröter et al., 2026). Outside kidney transplantation, studies in pediatric and hematologic populations have also reported poorer crude outcomes with aspergillosis than with PJP, a pattern directionally consistent with our findings, although differences in host populations and study designs limit direct comparability (Steingrímsson et al., 2020; Niyomthammarat et al., 2026). Our study extends this evidence by comparing PJP and IA within a common adjudication framework using consistent outcome definitions. However, the smaller IA group, the earlier onset distribution of IA, and the unadjusted nature of these analyses preclude attributing these differences to pathogen type itself.

We also evaluated acute rejection after IFI diagnosis. Previous studies have mainly examined rejection and CMV infection as factors preceding PJP or IFI (Hosseini-Moghaddam et al., 2018; Seok et al., 2020a; Cheng et al., 2024; Eberl et al., 2024), whereas evidence concerning acute rejection after IFI diagnosis is limited. In the matched analysis, IFI was associated with a higher risk of acute rejection after the index date. In the comparison of PJP and IA, rejection occurred in 12 PJP recipients and in none of the IA recipients, precluding HR estimation; the more frequent occurrence of death or graft failure among recipients with IA may have shortened the period during which rejection could be observed. Infection severity and treatment-related immunosuppression changes may also contribute. The relationship between temporary immunosuppression reduction and outcomes during severe infection may vary by organ type and clinical context (Rivet et al., 2025; Vaidie et al., 2025). Antifungal drug interactions and organ toxicity further require infection control and immunosuppression management to be assessed in parallel (Fishman et al., 2019; Husain and Camargo, 2019). A zero-event contrast should not be interpreted as evidence that a pathogen protects against or causes rejection. Clinically, IFI diagnosis may warrant closer short-term surveillance of graft function and rejection during infection treatment, with immunosuppression individualized according to infection severity, graft status, and treatment-related drug interactions.

Strengths of this study include the use of standardized IFI definitions with clinical case adjudication, explicit incorporation of infection timing in the primary time-varying analysis, and risk-set matching for temporal validation. We also evaluated acute rejection as a secondary outcome and characterized post-IFI outcomes according to onset timing and IFI type. Limitations include the single-center retrospective design and its associated potential for residual confounding and limited generalizability; possible selection bias related to incomplete follow-up among recipients who left the center within the first post-transplant year; potential ascertainment bias from clinically indicated rather than routine fungal testing, with possible under ascertainment of subclinical IFI; incomplete longitudinal characterization of infection severity, antifungal treatment, and changes in immunosuppression; inability to reliably reconstruct individual prophylaxis adherence, dose modification, and discontinuation; sparse events for some pathogen-specific outcomes; and longitudinal laboratory measurements that were obtained according to clinical need and were affected by missing data. These findings should be validated in multicenter cohorts with different pathogen distributions and diagnostic practices.

## 5 Conclusion

Post-transplant IFI was associated with higher risks of the composite outcome of all-cause mortality or graft failure and acute rejection in kidney transplant recipients. Within-IFI analyses showed poorer post-IFI outcomes with early versus late IFI and with IA versus PJP. These findings support coordinated attention to infection control, graft function, and immunological monitoring after IFI diagnosis, with consideration of onset timing and IFI type.

## Data Availability

The raw data supporting the conclusions of this article will be made available by the authors, without undue reservation.
