# Supplementary material for: Do invasive fungal infections affect kidney transplantation outcomes?
Source: Front Cell Infect Microbiol. 2026 Oct 27;16:1982586. doi: 10.3389/fcimb.2026.1982586 (PMC13747841; doi:10.3389/fcimb.2026.1982586)
Supplement: Supplementary file 1 [file Table1.docx]

# Supplementary Material

**Supplementary Table S1. Cohort selection and follow-up completeness.**

| Characteristic | Included in analysis cohort  (n = 752) | Not included in analysis cohor  t (n = 660) | Absolute standardized difference |
| --- | --- | --- | --- |
| Transplant year |  |  |  |
| 2020 | 103 (13.7%) | 155 (23.5%) | 0.25 |
| 2021 | 175 (23.3%) | 181 (27.4%) | 0.10 |
| 2022 | 215 (28.6%) | 199 (30.2%) | 0.03 |
| 2023 | 259 (34.4%) | 125 (18.9%) | 0.36 |
| Follow-up duration, median [IQR], days | 937 [682–1,254] | 68 [28–128] | 2.61 |
| Follow-up end reason |  |  |  |
| Administrative censoring | 525 (69.8%) | 0 (0.0%) | 2.15 |
| Loss to follow-up | 143 (19.0%) | 660 (100.0%) | 2.92 |
| Graft failure | 32 (4.3%) | 0 (0.0%) | 0.30 |
| Death | 52 (6.9%) | 0 (0.0%) | 0.39 |

**Supplementary Table S2. Clinical characteristics of PJP cases.**

| Characteristic | PJP cases (n = 99) |
| --- | --- |
| Symptoms and Signs |  |
| Fever | 78 (78.8%) |
| Cough | 73 (73.7%) |
| Dyspnea | 65 (65.7%) |
| Sputum production | 46 (46.5%) |
| Hypoxemia | 94 (94.9%) |
| Radiographic Patterns on computed tomography |  |
| Bilateral ground-glass opacities | 98 (99.0%) |
| Pulmonary consolidation | 37 (37.4%) |
| Inflammatory nodules | 22 (22.2%) |
| Pleural effusion | 4 (4.0%) |
| Microbiologic Evidence |  |
| Respiratory mNGS positive | 96 (97.0%) |
| Specimen Type |  |
| Bronchoalveolar lavage (BAL) | 93 (93.9%) |
| Sputum | 3 (3.0%) |
| Serum β-D-glucan, pg/mL | 351.2 [154.6–603.1] |
| Disease Severity and treatment |  |
| PaO₂/FiO₂ at diagnosis, mm Hg | 316.5 [215.5–391.8] |
| Oxygen and Ventilatory Support (highest level within 72 h) |  |
| No supplemental oxygen | 3 (3.0%) |
| Conventional oxygen | 17 (17.2%) |
| High-flow nasal cannula | 19 (19.2%) |
| Noninvasive ventilation | 49 (49.5%) |
| Invasive mechanical ventilation | 11 (11.1%) |
| ICU admission | 20 (20.2%) |
| Acute kidney injury | 18 (18.2%) |
| TMP-SMX treatment | 99 (100.0%) |
| Initial TMP dose, mg/kg/day | 11.3 [8.8–13.8] |

**Supplementary Table S3. Clinical characteristics of IFI cases and risk-set matched controls.**

| Clinical characteristics | IFI cases (n = 137) | Matched control records (n = 548) | P value |
| --- | --- | --- | --- |
| Recipient characteristics |  |  |  |
| Recipient age, years | 39.0 [33.0–49.0] | 39.0 [32.0–48.0] | 0.920 |
| Recipient sex |  |  | 0.861 |
| Male | 91 (66.4%) | 368 (67.2%) |  |
| Female | 46 (33.6%) | 180 (32.8%) |  |
| Body mass index, kg/m² | 21.8 [19.8–24.2] | 21.7 [19.6–24.4] | 0.771 |
| Hypertension history | 104 (75.9%) | 430 (78.5%) | 0.506 |
| Diabetes history | 14 (10.2%) | 69 (12.6%) | 0.391 |
| Chronic liver disease history | 17 (12.4%) | 111 (20.3%) | 0.011 |
| Coronary artery disease history | 6 (4.4%) | 24 (4.4%) | 1.000 |
| Cerebrovascular disease history | 4 (2.9%) | 18 (3.3%) | 0.793 |
| Smoking history | 34 (24.8%) | 117 (21.4%) | 0.359 |
| Alcohol history | 22 (16.1%) | 76 (13.9%) | 0.471 |
| Primary kidney disease |  |  | 0.715 |
| Glomerulonephritis | 23 (16.8%) | 111 (20.3%) |  |
| Diabetic kidney disease | 8 (5.8%) | 34 (6.2%) |  |
| Hypertensive nephropathy | 7 (5.1%) | 22 (4.0%) |  |
| Polycystic kidney disease | 1 (0.7%) | 7 (1.3%) |  |
| Alport syndrome | 0 (0.0%) | 1 (0.2%) |  |
| Other | 0 (0.0%) | 2 (0.4%) |  |
| Unknown | 98 (71.5%) | 371 (67.7%) |  |
| Dialysis modality |  |  | 0.113 |
| Hemodialysis | 104 (75.9%) | 461 (84.1%) |  |
| Hemodialysis + peritoneal dialysis | 4 (2.9%) | 10 (1.8%) |  |
| Peritoneal dialysis | 25 (18.2%) | 68 (12.4%) |  |
| Preemptive transplantation | 4 (2.9%) | 9 (1.6%) |  |
| Dialysis duration, months | 24.0 [13.0–41.0] | 26.0 [15.0–43.0] | 0.482 |
| Transplant and perioperative characteristics |  |  |  |
| Repeat kidney transplantation | 1 (0.7%) | 4 (0.7%) | 1.000 |
| Operation duration, min | 166.0 [143.0–190.0] | 165.0 [141.0–187.0] | 0.819 |
| Delayed graft function | 32 (23.4%) | 90 (16.4%) | 0.056 |
| Perioperative reoperation | 18 (13.1%) | 22 (4.0%) | <0.001 |
| Urine leak | 10 (7.3%) | 11 (2.0%) | 0.006 |
| Lymphatic leak | 7 (5.1%) | 7 (1.3%) | 0.020 |
| Wound-healing disorder | 4 (2.9%) | 8 (1.5%) | 0.293 |
| Graft ureteral stenosis | 9 (6.6%) | 25 (4.6%) | 0.335 |
| Perioperative hyperamylasemia | 29 (21.2%) | 69 (12.6%) | 0.013 |
| Perioperative hospital stay, days | 13.0 [9.0–24.0] | 11.0 [8.0–15.0] | <0.001 |
| Immunosuppression |  |  |  |
| Induction therapy |  |  | 0.518 |
| Basiliximab | 76 (55.5%) | 322 (58.8%) |  |
| ALG | 32 (23.4%) | 125 (22.8%) |  |
| rATG | 7 (5.1%) | 15 (2.7%) |  |
| Methylprednisolone alone | 22 (16.1%) | 86 (15.7%) |  |
| Initial maintenance regimen |  |  | 0.039 |
| Tacrolimus + MPA + prednisone | 136 (99.3%) | 539 (98.4%) |  |
| Other regimen | 1 (0.7%) | 9 (1.6%) |  |
| Immunologic and donor characteristics |  |  |  |
| HLA mismatch points | 3.0 [3.0–4.0] | 3.0 [3.0–4.0] | 0.763 |
| Donor age, years | 52.0 [45.0–59.0] | 49.0 [39.0–56.0] | <0.001 |
| Donor sex |  |  | 0.787 |
| Male | 114 (83.2%) | 451 (82.3%) |  |
| Female | 23 (16.8%) | 97 (17.7%) |  |
| Donor type |  |  | 0.273 |
| DBD | 133 (97.1%) | 542 (98.9%) |  |
| DCD | 2 (1.5%) | 1 (0.2%) |  |
| Living donor | 2 (1.5%) | 5 (0.9%) |  |
| Expanded-criteria donor | 51 (38.6%) | 165 (30.8%) | 0.112 |
| Donor serum creatinine, μmol/L | 107.5 [77.0–145.0] | 98.0 [67.0–145.0] | 0.822 |
| Donor body mass index, kg/m² | 22.9 [20.8–24.8] | 23.4 [21.4–25.4] | 0.027 |

**Supplementary Table S4. Factors associated with the 1-year composite outcome of all-cause mortality or graft failure in the risk-set matched cohort.**

| Variable | Univariable hazard ratio (95% CI) | Univariable P value | Multivariable hazard ratio (95% CI) | Multivariable P value |
| --- | --- | --- | --- | --- |
| Index exposure |  |  |  |  |
| IFI | 11.20 (6.26–20.04) | <0.001 | 10.83 (5.51–21.29) | <0.001 |
| Recipient characteristics |  |  |  |  |
| Recipient age, per 10 years | 1.17 (0.90–1.52) | 0.234 | 0.91 (0.64–1.30) | 0.616 |
| Recipient sex |  | 0.543 |  |  |
| Male | Reference |  |  |  |
| Female | 1.22 (0.65–2.30) | 0.543 |  |  |
| Body mass index, per 5 kg/m² | 1.07 (0.74–1.54) | 0.727 |  |  |
| Hypertension history | 0.57 (0.29–1.10) | 0.093 |  |  |
| Diabetes history | 2.49 (1.16–5.35) | 0.020 | 3.79 (1.11–12.93) | 0.034 |
| Coronary artery disease history | Not estimable (separation) |  |  |  |
| Cerebrovascular disease history | 2.13 (0.87–5.19) | 0.096 |  |  |
| Smoking history | 0.86 (0.39–1.89) | 0.701 |  |  |
| Alcohol history | 1.29 (0.56–2.97) | 0.555 |  |  |
| Primary kidney disease | Not estimable (separation) |  |  |  |
| Dialysis modality |  |  |  |  |
| Hemodialysis | 0.38 (0.20–0.74) | 0.004 |  |  |
| Hemodialysis +  peritoneal dialysis | 0.97 (0.19–4.98) | 0.970 |  |  |
| Peritoneal dialysis | 2.93 (1.50–5.75) | 0.002 |  |  |
| Preemptive transplantation | 1.53 (0.37–6.41) | 0.559 |  |  |
| Dialysis duration, per month | 1.00 (0.99–1.01) | 0.576 |  |  |
| Transplant and perioperative characteristics |  |  |  |  |
| Repeat kidney transplantation | 1.24 (0.15–10.53) | 0.846 |  |  |
| Operation duration, per 10 minutes | 1.07 (1.00–1.15) | 0.046 |  |  |
| Delayed graft function | 1.74 (0.84–3.63) | 0.137 | 1.13 (0.44–2.89) | 0.796 |
| Perioperative reoperation | 3.76 (1.46–9.71) | 0.006 |  |  |
| Urine leak | 0.97 (0.23–4.12) | 0.965 |  |  |
| Lymphatic leak | 12.00 (1.66–86.94) | 0.014 |  |  |
| Wound-healing disorder | 1.00 (0.14–7.10) | 1.000 |  |  |
| Graft ureteral stenosis | 1.35 (0.38–4.76) | 0.639 |  |  |
| Perioperative hyperamylasemia | 2.06 (0.99–4.30) | 0.054 |  |  |
| Perioperative hospital stay, per day | 1.01 (1.00–1.03) | 0.050 |  |  |
| Immunosuppression |  |  |  |  |
| Induction therapy |  |  |  |  |
| Basiliximab | 1.49 (0.77–2.87) | 0.233 |  |  |
| ALG | 0.31 (0.12–0.84) | 0.020 |  |  |
| rATG | 1.00 (0.14–7.10) | 1.000 |  |  |
| Methylprednisolone alone | 1.72 (0.68–4.31) | 0.249 |  |  |
| Immunologic and donor characteristics |  |  |  |  |
| HLA mismatch points, per point | 1.09 (0.89–1.32) | 0.416 |  |  |
| Donor age, per 10 years | 1.61 (1.24–2.09) | <0.001 | 1.25 (0.95–1.65) | 0.107 |
| Donor sex |  | 0.319 |  |  |
| Male | Reference |  |  |  |
| Female | 1.41 (0.72–2.74) | 0.319 |  |  |
| Donor type | Not estimable (separation) |  |  |  |
| Expanded-criteria donor | 2.10 (1.04–4.23) | 0.039 |  |  |
| Donor serum creatinine, per 50 μmol/L | 1.10 (0.87–1.40) | 0.411 |  |  |
| Donor body mass index, per 5 kg/m² | 0.89 (0.55–1.43) | 0.624 |  |  |
| Time-anchored rejection and infection exposures: 180-day window |  |  |  |  |
| Acute rejection within 180 days before index | 4.00 (1.58–10.11) | 0.003 | 2.48 (0.49–12.66) | 0.274 |
| Respiratory bacterial infection within 180 days before index | 2.05 (0.58–7.25) | 0.265 | 5.58 (1.17–26.60) | 0.031 |
| Respiratory viral infection within 180 days before index | 0.68 (0.19–2.47) | 0.562 |  |  |
| CMV positivity within 180 days before index | Not estimable (separation) |  |  |  |
| BK viruria within 180 days before index | 4.00 (0.40–39.83) | 0.237 |  |  |

Abbreviations: ALG, anti-lymphocyte globulin; BK, BK polyomavirus; CI, confidence interval; CMV, cytomegalovirus; HLA, human leukocyte antigen; HR, hazard ratio; IFI, invasive fungal infection; rATG, rabbit antithymocyte globulin.

# Supplementary Figures and figure legends

**Supplementary Figure S1. Distribution of time to first IFI after kidney transplantation.**

**Supplementary Figure S2. Adjusted time-varying hazard ratios for time-updated IFI status and component outcomes.**

(A) All-cause mortality; (B) Death-censored graft failure; and (C) Acute rejection. Panels A–C present cause-specific hazard ratios with outcome-specific censoring: graft failure for all-cause mortality, death for death-censored graft failure, and death or graft failure for acute rejection. All models were adjusted for recipient age, history of diabetes, donor age, and delayed graft function.

**Supplementary Figure S3. Cumulative incidence of outcomes during 1-year follow-up after assigned index dates in IFI cases and risk-set matched controls.**

(A) All-cause mortality, with graft failure as the competing event. (B) Death-censored graft failure, with death as the competing event. (C) Acute rejection, with death and graft failure as competing events.

**Supplementary Figure S4. Cumulative probability of the composite outcome of all-cause mortality or graft failure after first IFI diagnosis, according to onset timing and IFI type.**

(A) Early versus late IFI; (B) PJP versus IA. Curves show 1−Kaplan–Meier estimates over 365 days after IFI diagnosis. Comparisons were based on Cox models.

**Supplementary Figure S5. Observed longitudinal core laboratory values around first IFI diagnosis: early versus late IFI. Points and error bars indicate medians and interquartile ranges.**

**Supplementary Figure S6. Observed longitudinal systemic laboratory values around first IFI diagnosis: early versus late IFI. Points and error bars indicate medians and interquartile ranges.**

**Supplementary Figure S7. Observed longitudinal core laboratory values around first IFI diagnosis: PJP versus IA. Points and error bars indicate medians and interquartile ranges.**

**Supplementary Figure S8. Observed longitudinal systemic laboratory values around first IFI diagnosis: PJP versus IA. Points and error bars indicate medians and interquartile ranges.**
